# Defect Passivation and Enhanced Hole Extraction in Inverted Perovskite Solar Cells via CeO_2_@MoS_2_ Interfacial Engineering

**DOI:** 10.3390/nano16030188

**Published:** 2026-01-30

**Authors:** Pradeep Kumar, Chia-Feng Li, Hou-Chin Cha, Yun-Ming Sung, Yu-Ching Huang, Kuen-Lin Chen

**Affiliations:** 1 Department of Materials Engineering, Ming Chi University of Technology, New Taipei City 243303, Taiwan; pksingh0021@mail.mcut.edu.tw (P.K.);; 2 Department of Materials Science and Engineering, National Taiwan University, Taipei 106319, Taiwan; 3 College of Engineering, Ming Chi University of Technology, New Taipei City 24301, Taiwan; hccha@mail.mcut.edu.tw; 4 Organic Electronics Research Center, Ming Chi University of Technology, New Taipei City 243303, Taiwan; 5 Department of Physics, National Atomic Research Institute, Taoyuan City 325207, Taiwan; 6 Center for Plasma and Thin Film Technologies, Ming Chi University of Technology, New Taipei City 243303, Taiwan; 7 Department of Physics, National Chung Hsing University, Taichung 402, Taiwan

**Keywords:** perovskite solar cells, hole transport layer, interfacial engineering, CeO_2_@MoS_2_ nanocomposite, defect passivation

## Abstract

Nanomaterial-based hole transport layers (HTLs) play a vital role in regulating interfacial charge extraction and recombination in perovskite solar cells (PSCs). To improve PSC efficiency, hydrothermally synthesized CeO_2_@MoS_2_ nanocomposites (CM NCs) were incorporated as an interfacial buffer layer into a NiO_X_/MeO-2PACz HTL. The introduction of CM NCs induces strong interfacial interactions, where Mo sites in MoS_2_ interact with NiO_X,_ modulating the Ni^2+^/Ni^3+^ ratio and reducing the interfacial trap density. Moreover, CeO_2_ promotes the formation of oxygen vacancies, collectively improving the conductivity and hole transport capability of the NiO_X_ HTL. The MoS_2_-grafted CeO_2_ interlayer effectively tailors the interfacial energetics and creates an effective channel for hole transfer, thereby reducing open-circuit voltage (V_OC_) loss and enhancing device performance. This interface modification efficiently enhances hole extraction, and non-radiative recombination is effectively suppressed at the NiO_X_/MeO-2PACz/perovskite interface. Thereby, incorporating 2 vol% CM NCs into PSCs achieved a power conversion efficiency (PCE) of 17.93%, compared to 17.50% for a 1 vol% CM NCs-based device and 17.01% for the unmodified control device. The enhanced performance at the optimized CM NCs concentration is attributed to effective defect passivation, reduced V_OC_ loss, and improved interfacial band alignment, which together facilitate hole extraction and suppress non-radiative recombination. However, excessive CM NCs incorporation (4 vol%) leads to increased interfacial resistance, partial hole blocking effects associated with the n-type nature of CeO_2_, and aggravated recombination, resulting in degraded device performance. These results demonstrate that precise control over CM NCs interlayer thickness and concentration is critical for maximizing device performance, providing a robust strategy for designing high-efficiency and stable NiO_X_-based PSCs and advancing nanocomposite-enabled interfacial engineering for photovoltaic applications.

## 1. Introduction

Nanotechnology advancements have opened new avenues to improve the performance of solar energy utilization. The use of nanomaterials in the energy sector has attracted significant attention due to their exceptional optical, electrical, and catalytic properties, offering promising pathways for advancing energy conversion and storage technologies [1]. Nanomaterials can significantly enhance the absorbance of solar light; therefore, their use in solar cells has become prevalent to improve power conversion efficiency (PCE) [2]. Organic solar cells (OSCs) have recently demonstrated PCE exceeding 20% [3]. A typical single-junction OSC comprises a photoactive layer sandwiched between an electron transport layer (ETL) and a hole transport layer (HTL). Nevertheless, poor operational stability continues to impede commercialization, largely due to the limited insight into the fundamental degradation phenomena in OSCs. Therefore, perovskite solar cells (PSCs) have largely overcome these limitations and have achieved PCE exceeding 25% [4,5]. To fabricate a PSC, several layers are assembled in sequence, including a transparent conductive substrate (TCS), an ETL, a perovskite light-absorbing layer, an HTL, and finally a metal contact electrode. Furthermore, optimizing each functional layer, while keeping charge mobility, electron-hole balance, and optical transmittance, is essential for the fabrication of high-performance n–i–p (regular planar) and p–i–n (inverted planar) hybrid perovskite solar cell architectures. The inverted (p–i–n) architecture has attracted considerable interest due to its reduced hysteresis, compatibility with low-temperature processing, and suitability for monolithic tandem integration with silicon, Cu(In, Ga)Se_2_ (CIGS), and Sn–Pb perovskite subcells. Recently, p–i–n perovskite solar cells have achieved certified power conversion efficiencies exceeding 27%, demonstrating their strong technological potential [6].

The efficiency and operational stability of PSCs are significantly influenced by the engineering and selection of ETL and HTL materials. Accordingly, numerous studies have focused on ETL modification and its influence on PSC performance [7]. Nevertheless, the HTL plays an essential role in selectively extracting photogenerated holes from the perovskite absorber and efficiently transports them to the metal contact electrode [8]. In PSCs, the open-circuit voltage (V_OC_) originates from the splitting of the electron and hole quasi-Fermi levels; however, non-radiative recombination of excess free charge carriers leads to a reduction in this splitting [9]. Numerous non-radiative recombination processes in PSCs have been reported, including trap-assisted recombination, carrier-carrier scattering, and Auger recombination [10,11]. Nonradiative recombination at the interfaces of adjacent functional layers plays a crucial role in determining charge extraction and transport, which is a major factor limiting V_OC_ and overall device efficiency [12]. Consequently, the HTL in PSCs has been widely investigated to improve the interfacial properties, prevent non-radiative recombination, and facilitate efficient charge-carrier transport. To this end, various nanomaterials have been employed as HTLs in PSCs, such as NiO_X_, CuO_X_, CuS, MoS_2_, VO_X_, WO_3_, and CuSCN [13]. Recently, NiO_X_ has been extensively employed as an HTL material in PSCs due to its multiple benefits, such as low cost, ease of fabrication, good stability, and favourable band level structure [14]. However, multiple approaches have been employed to enhance the performance of NiO_X_, including the incorporation of metal ions and small molecules as dopants. To increase the ratio of Ni^3+^/Ni^2+^, various metals are doped in NiO_X,_ including alkali metal and alkaline earth metal ions (Li^+^, K^+^, Rb^+^, Mg^2+^) [15,16], transition metal ions (Cu^2+^, Fe^2+^) [16,17], and rare earth metal ions (Ce^4+^, Yb^3+^) [18]. However, this approach introduces new challenges, such as lattice distortion [19] and reduced transmittance of NiO_X_ HTL. Therefore, alternative modification strategies have been explored, such as incorporating carbon quantum dots [20], 3,6-difluoro-2,5,7,7,8,8-hexacyanoquinodimethane (F2HCNQ) [21], 2,2′-(perfluoronaphthalene-2,6-diylidene) dimalononitrile (F6TCNNQ) [22], which possess high electron affinity. These additives effectively suppress charge-carrier recombination and ultimately improve hole-transport performance. In 2014, K.-C. Wang et al. reported NiO_x_ nanocrystals as p-type electrodes for PSCs, achieving a PCE of 9.51% using NiO_x_/perovskite/PCBM device configuration [23].

More recently, p-type NiO_x_ layer modified with self-assembled monolayer (SAM), such as 2PACz, MeO-2PACz, Me-4PACz, or their mixtures, has emerged as an ideal hole-transport structure in PSCs [24,25,26], as it reduces interface trap density and enhances hole-selectivity by increasing the work function [27]. The work-function enhancement through SAM modification is ascribed to a vacuum-level shift, originating from the intrinsic dipole moment of the SAM molecule, and establishment of a space-charge region at the NiO_x_/SAM interface [27]. Therefore, upward band bending near the interface facilitates hole extraction and charge transport. To date, PSCs employing inorganic HTL generally exhibit lower PCEs due to detrimental interfacial reaction, which impedes the hole transport and increases carrier recombination rate at the interface [28]. Basically, the Ni^3+^ cations in NiO_x_ HTL can interact with SAM molecules or perovskite materials, which reduces the amount of Ni^3+^ cations in NiO_x_. This reduction leads to a decreased electrical conductivity of the NiO_x_ HTL and, consequently, a lower PCE in PSCs [29]. Introducing nanomaterial interlayers between the HTL and SAM has emerged as an efficient approach, leading to significant improvement in the fill factor (FF) and enhanced hole mobility. The FF value can be boosted from 0.22 to 0.80, accompanied by an increase in hole mobility from 10^−6^ to 10^−4^ cm^2^V^−1^s^−1^ [30].

CeO_2_ has abundant oxygen vacancies and demonstrates significant oxygen storage and release capability through the Ce^4+^/Ce^3+^ redox cycle [31]. The interfacial interaction between NiO_x_ and CeO_2_ can generally increase oxygen-vacancy concentration and encourage oxygen diffusion, which contributes to superior performance in a solar cell [32,33]. Solar cell efficiency has been improved by creating an interface using MoS_2_ with TiO_2_, WS_2_, graphene, etc. [34]. Shanmugan et al. demonstrated a solar cell efficiency of 1.8% by creating a Schottky junction at the MoS_2_@Au interface [35]. In 2022, Yaohui Li et al. [36] enhanced the PCE of PSCs by modifying the HTL with MoS_2_-doped poly(3,4-ethylenedioxythiophene) (PEDOT). Hui Huang et al. [37] achieved 7.24% PCE using MoS_2_-doped PEDOT:PSS.

In this work, hydrothermally synthesized CeO_2_@MoS_2_ nanocomposites (CM NCs) were introduced as an interfacial modifier to augment the PCE of p–i–n PSCs with a glass/FTO/NiO_x_/MeO-2PACz/perovskite/PC_61_BM/BCP/Ag device architecture. An ultrathin CeO_2_@MoS_2_ nanocomposite layer was inserted between NiO_x_ and MeO-2PACz. The oxygen vacancies and defect sites in CeO_2_@MoS_2_ promote hole extraction and suppress interfacial non-radiative recombination, thereby prolonging carrier lifetime and enhancing PCE.

## 2. Experimental Method

### 2.1. Reagents and Materials

Cerium (III) nitrate hexahydrate (Ce(NO_3_)_3_·6H_2_O), L-cysteine, and sodium molybdate (Na_2_MoO_4_) were obtained from Sigma-Aldrich (St. Louis, MO, USA). Nickel oxide nanoparticles (NPs) and PC_61_BM (99.0%) were obtained from Lumtec (New Taipei City, Taiwan). Ethanol (EtOH, >99%) was purchased from Fisher Chemical (Waltham, MA, USA). Dimethyl sulfoxide (DMSO, >99.9%), chlorobenzene (CB, >99.0%), and isopropyl alcohol (IPA, 99.8%) were obtained from Acros Organics (Geel, Belgium). Rubidium iodide (RbI), 2,2,2-Trifluoroethanol (TFE), Cesium iodide (CsI), and Dimethylformamide (DMF, 99.8%) were purchased from Sigma-Aldrich. FTO glasses, Formamidinium Iodide (FAI), 2-Thiopheneethylammonium chloride (TEACl), and Formamidinium Bromide (FABr) were purchased from FrontMaterials (New Taipei City, Taiwan). Bathocuproine (BCP) and lead iodide (PbI_2_, 99.9985%) were obtained from TCI (Tokyo, Japan).

### 2.2. Synthesis of CeO_2_ Nanoparticles

**Figure 1:**
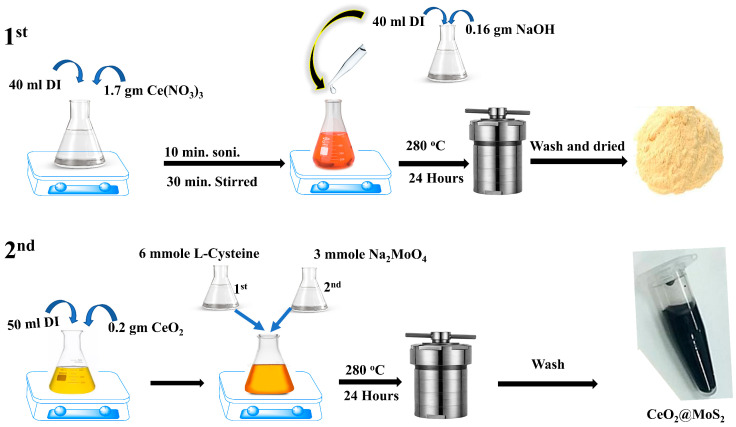
Schematic illustration of the synthesis process of the CM NCs.

CeO_2_ nanoparticles were synthesized via a hydrothermal method, as described in reference [38], with slight modifications. Briefly, 1.736 g of Ce(NO_3_)_3_·6H_2_O was dissolved in 40 mL of deionized (DI) water and stirred for 30 min, followed by 30 min of ultrasonication. The mixture was continuously stirred for a further 10 min. Separately, 0.16 g NaOH was dissolved in 40 mL of DI water to prepare a NaOH solution, and was subsequently added dropwise to the Ce precursor solution under continuous stirring (Figure 1), resulting in a final pH of 7.2. After stirring for 1 h, the mixture reached a pH of 4, was transferred into a 100 mL Teflon-lined stainless-steel autoclave, and heated at 180 °C for 24 h. The obtained white precipitate was separated by centrifugation, washed three times with DI water, and then dried for six hours at 60 °C in an oven to produce yellow CeO_2_ powder.

### 2.3. Synthesis of CeO_2_@MoS_2_ Nanocomposite

First, 0.2 g of CeO_2_ was immersed in 1 mL of DI water, stirred till a homogenous suspension was achieved, and then diluted with 35 mL of DI water. After 30 min of stirring, the liquid was ultrasonically stirred for a further 30 min. Subsequently, a solution of L-cysteine (6 mmol, 30 mL) was added, and the mixture was stirred for 30 min, after which a solution of Na_2_MoO_4_ (3 mmol, 15 mL) was added. The resulting solution was further stirred for 1 h, transferred into a 100 mL Teflon-lined stainless-steel autoclave, and heated at 280 °C for 24 h. After naturally cooling to room temperature, the obtained precipitate was collected by centrifugation and washed several times with DI water and ethanol. A schematic flowchart of the CM NC synthesis process is shown in Figure 1.

### 2.4. Device Fabrication

**Figure 2:**
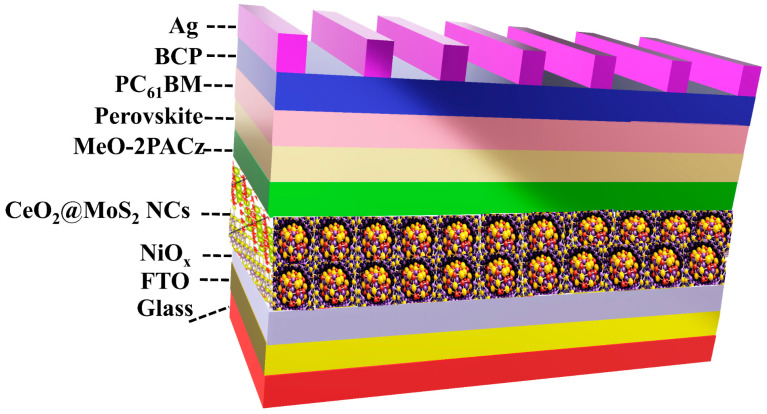
Schematic representation of the PSC device architecture.

PSCs were fabricated in a p–i–n configuration consisting of glass/FTO/NiO_x_/CM NCs/MeO-2PACz/perovskite/PC_61_BM/BCP/Ag, as schematically depicted in Figure 2. Before layer deposition, the FTO glass was cleaned using O_2_ plasma for 20 min. The NiO_x_ NPs HTL solution (5 mg/mL in DI water) was deposited by spin-coating at 5000 rpm for 20 s, followed by annealing at 150 °C for 20 min. The MeO-2PACz layer (3 mM in EtOH) was realized by spin-coating onto the prepared NiO_x_/FTO substrate at 3000 rpm for 20 s, and subsequently annealed at 100 °C for 5 min. The perovskite precursor solution was obtained by mixing CsI (0.42 M), FAI (0.35 M), and PbI_2_ (1.47 M) in a DMF:DMSO (4:1, *v*/*v*) solvent mixture, followed by stirring at room temperature for 8 h. The perovskite layer was deposited by spin coating the precursor solution onto the FTO/HTL substrate according to a previously reported procedure [39]. The TEACl solution (4 mM in IPA) was dropped onto the perovskite film, then spin-coated at 3000 rpm for 20 s and annealed at 150 °C for 40 s. The PC_61_BM electron transport layer (ETL) layer (20 mg/mL in CB) was spin-coated at 1000 rpm for 20 s. The BCP work function layer (0.5 mg/mL in TFE) was then deposited by spin-coating at 3000 rpm for 20 s. At last, the Ag electrode was deposited by thermal evaporation at 2 × 10^−6^ torr to complete device fabrication, yielding PCSs with an active area of 0.09 cm^2^. To enhance device performance, CM NCs were incorporated as an interfacial layer between the NiO_x_ and MeO-2PACz. A schematic representation of the glass/FTO/NiO_X_:CM NCs (25 nm)/MeO-2ACz/perovskite (550 nm)/PC_61_BM (50 nm)/BCP (5 nm)/Ag (120 nm) device with CM NCs is illustrated in Figure 2.

### 2.5. Characterization of Perovskite Film and Devices

The 1-Sun PCE of the PSCs was evaluated under AM1.5G solar simulator (Enlitech, SS-X100R AAA, Kaohsiung City, Taiwan). Transient photovoltage (TPV) and transient photocurrent (TPC) measurements were carried out using an all-in-one characterization platform (Paios, Fluxim AG, Winterthur, Switzerland) to examine charge-carrier recombination and extraction dynamics. The microstructure and lattice features of the CM NCs were investigated by high-resolution transmission electron microscopy (HRTEM, JEOL, Akishima, Japan, JEM-2100, LaB_6_ electron gun).

## 3. Results and Discussion

### 3.1. Characterization of CeO_2_@MoS_2_ Nanocomposites

**Figure 3:**
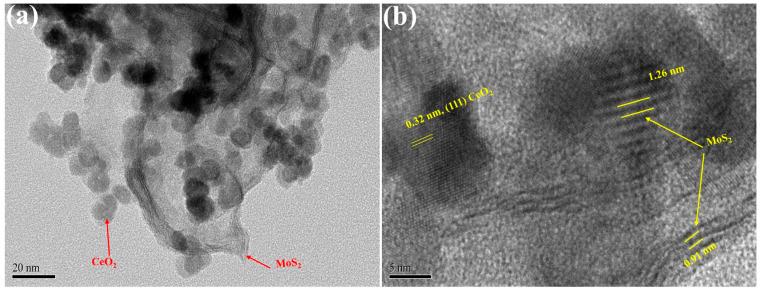
(**a**,**b**) High-magnification cross-section TEM images showing the morphology of CM NCs.

To confirm the internal structure, crystallinity, and defect characteristics of the nanomaterial, HRTEM analysis was performed. The results demonstrated that CeO_2_ nanoparticles are integrated with few-layer MoS_2_ nanosheets, forming a well-defined CeO_2_@MoS_2_ nanocomposite. As shown in Figure 3a,b, the CeO_2_ nanoparticles exhibit a roughly spherical morphology with a lattice spacing of 0.32 nm, corresponding to the (111) plane of cubic CeO_2_ [40]. Additionally, an ultrathin silk-like layered structure was observed on the surface of the CeO_2_ nanoparticles, attributed to few-layer MoS_2_ nanosheets, confirming the layered structure of MoS_2_. Expanded interlayer-like spacings of approximately 0.91 nm and 1.26 nm were observed at the CeO_2_–MoS_2_ interface, which are significantly larger than the typical (002) interlayer spacing (~0.62 nm) of pure MoS_2_ [41,42]. This expansion can be ascribed to lattice strain caused by lattice mismatch, strong interfacial interactions between CeO_2_ and MoS_2_, and the presence of sulfur vacancies and structural defects [43]. Furthermore, the oxygen-vacancy-rich CeO_2_ promotes interlayer expansion in MoS_2_, as indicated by the greater spacing of 1.26 nm in Figure 3b. These characteristics verify the production of few-layer, defect-rich MoS_2_ with increased interlayer spacing in the CM NCs. The multilayer MoS_2_ structure wrapping around CeO_2_ nanoparticles is expected to improve the structural stability and functional performance of CM NCs. Additionally, the TEM analysis revealed no secondary phases, demonstrating the excellent purity of the synthesized CM NCs.

**Figure 4:**
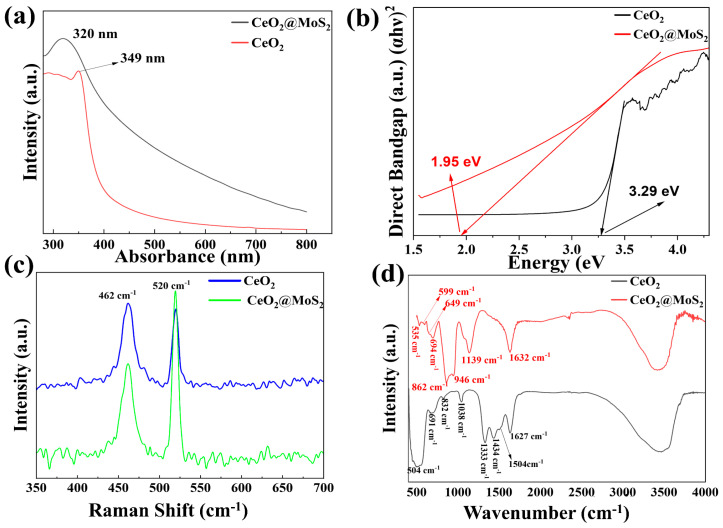
Optical properties of CeO_2_ and CM NCs at room temperature. (**a**) UV light absorbance spectra, (**b**) Direct bandgap calculated using Tauc plot, (**c**) Raman spectra, (**d**) FT−IR spectra.

The optical absorption properties of the synthesized materials were characterized using the UV-Vis spectrometer (JASCO U-550, Tokyo, Japan). As shown in Figure 4a, pure CeO_2_ exhibits a sharp absorbance peak at 349 nm [44,45]. Moreover, CM NCs possess a broad absorption spectrum, with a specific absorption peak at 320 nm, indicating a blue shift induced by coupling with MoS_2_. This behavior is attributed to strong interfacial interactions between CeO_2_ and MoS_2_, as well as quantum confinement effects and lattice distortion. The UV-Vis absorption spectra were used to draw Tauc plots as illustrated in Figure 4b, and the direct bandgap of CeO_2_ was determined to be 3.29 eV [46]. However, an effective bandgap of CM NCs was estimated to be 1.97 eV, which is significantly smaller than the direct bandgap of CeO_2_. The observed bandgap narrowing arises from electronic coupling between CeO_2_ and MoS_2_, as well as the formation of interfacial states and defect levels associated with oxygen vacancies and lattice distortions. Such bandgap narrowing is beneficial for enhanced visible-light harvesting and interfacial charge transfer between the HTL and the perovskite layer. The atomic vibrations and structural characteristics of the synthesized materials were investigated using Raman spectroscopy (NANOSCOPE/NS220-RM0121010, South Korea). In the Raman spectra, as shown in Figure 4c, two strong and sharp peaks appeared for both CeO_2_ and CeO_2_@MoS_2_. The Raman vibration at 462 cm^−1^ corresponds to the F_2g_ symmetric breathing mode of the Ce-O vibrational unit [47,48]. Additionally, the Raman band at 520 cm^−1^ is commonly attributed to the defect-induced vibrational bands in CeO_2_, associated with oxygen vacancies, lattice distortions, or surface defects [49]. Such defect-related vibrations are frequently enhanced in nanostructured materials due to their high surface-to-volume ratio. Moreover, the intensity of the 520 cm^−1^ peak is significantly enhanced in CeO_2_@MoS_2_, which can be attributed to an increased concentration of oxygen vacancies, interfacial charge transfer, and strain-induced symmetry breaking arising from strong CeO_2_–MoS_2_ interactions. These features indicate the presence of defect-rich CeO_2_ within the heterostructure. The Fourier transform infrared (FTIR) spectra were measured using a JASCO-FT-IR 4600 spectrometer, Tokyo, Japan. Figure 4d illustrates the FTIR spectra of CeO_2_ and CM NCs. Eight characteristic molecular vibrations were observed for CeO_2_ at 504, 691, 832, 1038, 1333, 1434, 1504, and 1627 cm^−1^. The bands at 504 and 691 cm^−1^ were attributed to the Ce-O stretching vibrations, confirming the formation of the CeO_2_ structure [50,51]. The bands at 832 cm^−1^ and 1038 cm^−1^ were observed due to the metal-oxygen bonding [52] and Ce–O–Ce bridging vibrations [53], respectively. Most published studies reported that CeO_2_ NPs readily react with CO_2_ in air, resulting in the formation of carbonate species absorbed on the CeO_2_ surface [54,55]. However, R. Bakkiyaraj et al. [53] and Bhawana Jain et al. [56] reported that molecular vibration in the range 1500–1380 cm^−1^ and 1396–1541 cm^−1^, respectively, corresponded to physically absorbed water molecules. Accordingly, the peaks at 1333, 1434, and 1504 cm^−1^ are attributed to physically adsorbed water and/or overlapping carbonate-related vibrational modes on the CeO_2_ surface. In addition, the broad bands in the 3750–3000 cm^−1^ region and the band at 1627 cm^−1^ are attributed to physically bonded water and various surface hydroxyl groups, corresponding to O–H stretching and bending vibrations, respectively [57]. On the other hand, CM NCs exhibited pronounced spectral changes, confirming successful bonding and strong interfacial interactions. The Ce-O stretching vibrations evolve into several peaks at 535, 599, 694, and 862 cm^−1^, indicating lattice deformation, increased oxygen vacancy concentration, and significant CeO_2_-MoS_2_ interaction. The vibration bands at 649 cm^−1^ [58,59] and 946 cm^−1^ [60] are attributed to Mo–S and Mo–O–Ce interfacial vibrations, respectively, indicating chemical bonding rather than physical mixing. Additionally, the band at 1139 cm^−1^ corresponds to sulfur vacancy-related states in MoS_2_ and defect-induced vibrational modes. Compared with pure CeO_2_, the enhanced intensity of the band 1632 cm^−1^, corresponding to adsorbed water bending vibrations, indicates higher surface activity and defect density in the CM NCs. Overall, the observed peak shifts, splitting, and emergence of new vibrational modes in the CM NCs collectively demonstrate strong interfacial interactions, defect enrichment, and the formation of oxygen and sulfur vacancies, consistent with the Raman and TEM results. These structural features are expected to facilitate improved charge transfer and enhanced optical and catalytic performance.

### 3.2. Interfacial Morphology Optimization of NiO_X_ via CM NC Incorporation

**Figure 5:**
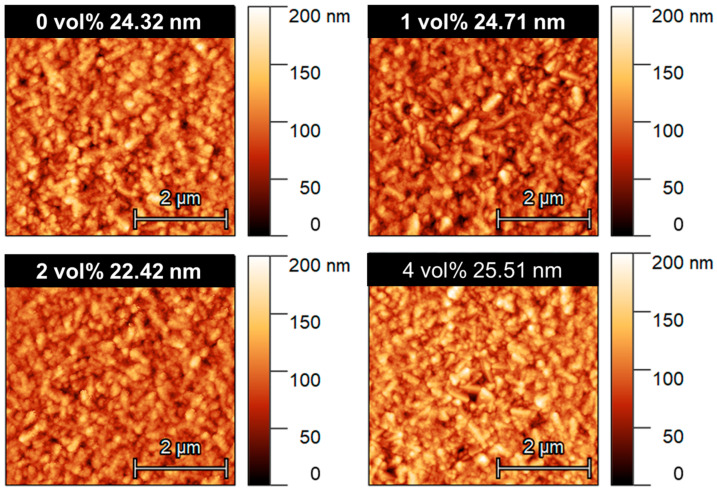
AFM image of NiO_X_ film incorporating different concentrations of CM NCs.

In addition, the morphology and surface roughness of the NiO_X_ layer are critical factors influencing the PCE of PSCs. Atomic force microscope (AFM) analysis was employed to investigate the surface characteristics of NiO_X_ films with varying CM NCs concentrations (Figure 5). As shown in the images, the pristine NiO_X_ film (0 vol%) exhibited a root-mean-square (RMS) roughness of 24.32 nm. With the incorporation of 2 vol% CM NCs, the film achieved the most uniform surface with a minimum RMS value of 22.42 nm, indicating that an optimal concentration of CM NCs can effectively smooth the NiO_X_ surface. However, increasing the concentration further to 4 vol% led to a higher RMS value of 25.51 nm, potentially due to the slight aggregation of nanocrystals at higher loading levels. The reduced surface roughness at 2 vol% was beneficial for ensuring intimate physical contact and carrier extraction at the interface between the NiO_X_ and the subsequent perovskite active layer, which is essential for maximizing device performance.

### 3.3. Device Performance of Perovskite Solar Cells

**Figure 6:**
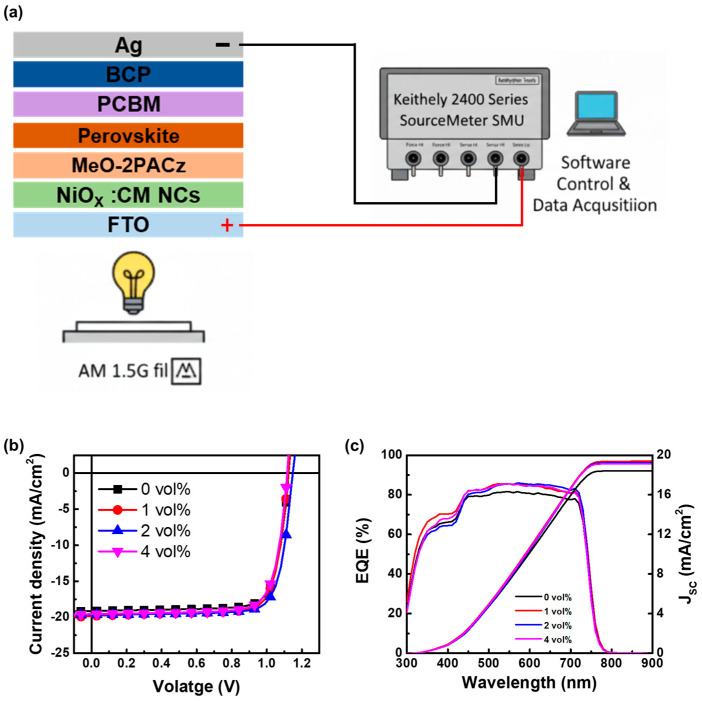
(**a**) Schematic illustrations of the PSCs device architecture and the electrical measurement setup. (**b**) J–V curves and (**c**) EQE spectra with integrated J_SC_ of PSCs incorporating different concentrations of CM NCs.

**Figure 7:**
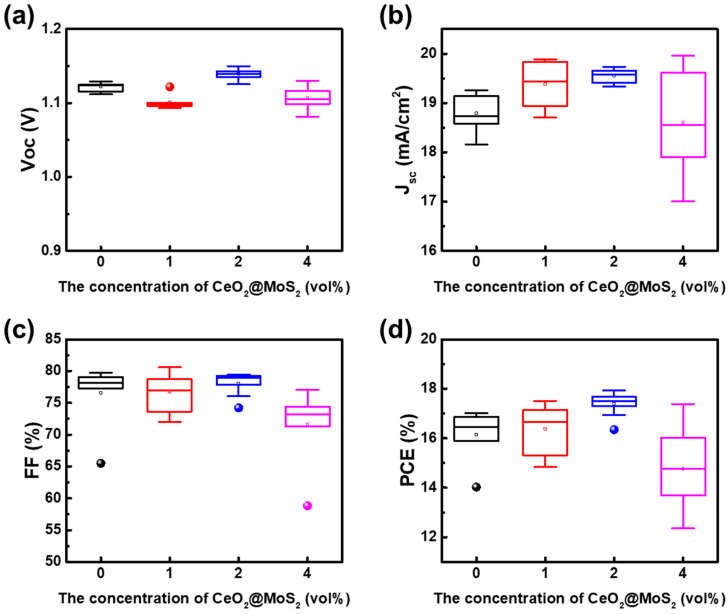
Device characteristics of PSCs as a function of CM NCs concentrations in the NiO_x_ NPs solution. (**a**) V_OC_, (**b**) J_SC_, (**c**) FF, and (**d**) PCE.

In this study, PSCs with a p–i–n device structure of glass/FTO/NiO_x_ NPs/MeO-2PACz/perovskite/PC_61_BM/BCP/Ag were fabricated to investigate the effect of incorporating CM NCs into the NiO_x_ HTL. CeO_2_ is well known for its high oxygen storage and release capacity through the reversible Ce^4+^/Ce^3+^ redox cycle, which produces a high density of oxygen vacancies [31]. Here, the interfacial interaction between NiO_x_ and CeO_2_ further increased the concentration of oxygen vacancies and enhanced oxygen diffusion, thereby improving the electrical properties of the HTL. To evaluate the influence of the CM NCs layer on device performance, PSCs incorporating different concentrations of CM NCs were systematically investigated. Figure 6a shows the schematic diagram of the PSCs device structure and the electrical measurement layout. The J-V curves and EQE spectra with integrated J_SC_ of PSCs incorporating different concentrations of CM NCs are presented in Figure 6b,c, respectively. The device characterizations and J-V curves with different concentrations of CM NCs are listed in Table 1. The photovoltaic characteristics of these devices as a function of CM NCs concentration under 1-sun illumination are shown in Figure 7. The control device without CM NCs exhibited an average V_OC_ of 1.12 V, an average J_SC_ of 18.79 mA/cm^2^, and an average FF of 76.59%, resulting in an average PCE of 16.14%. The perovskite solar cells performed better after adding 2 vol% CM NCs, with an average PCE of 17.40%, a V_OC_ of 1.14 V, a J_SC_ of 19.56 mA cm^−2^, and an FF of 78.07%. However, further increasing the CM NCs concentration to 4 vol% led to a deterioration in device performance. The performance decline suggested that excessive CM NCs may have disrupted the dispersion of the NiO_x_ NPs solution, inducing NiO_x_ NPs aggregation and increasing charge-transport resistance. To corroborate the J_SC_ enhancement, EQE measurements were conducted on perovskite solar cells fabricated with NiO_X_ films containing different CM NCs concentrations. As shown in Figure 6c, an increase in CM NCs concentration yields a progressive enlargement of the integrated EQE area, leading to higher simulated current densities that closely match the measured J_SC_ values. Based on these results, 2 vol% of CM NCs was identified as the optimal concentration for enhancing charge extraction and improving PSC efficiency.

**Figure 8:**
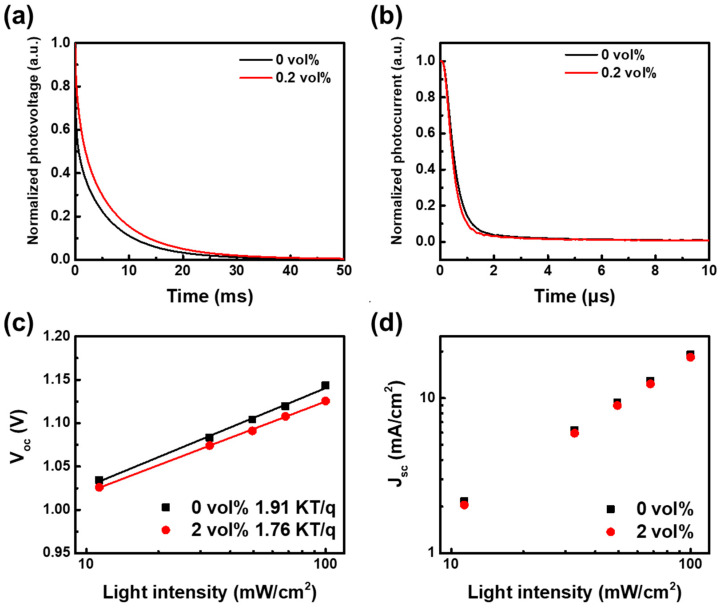
Charge-carrier dynamics of PSCs with 0 vol% and 2 vol% CM NCs. (**a**) normalized TPV. (**b**) normalized TPC, and light-intensity dependence of (**c**) V_OC_ and (**d**) J_SC_.

**Figure 9:**
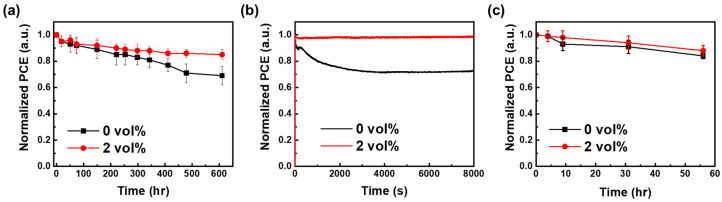
(**a**) Long-term stability of PSCs with and without CeO_2_@MoS_2_ interlayer under N_2_ atmosphere. (**b**) MPP tracking of PSCs with and without CeO_2_@MoS_2_ under N_2_ atmosphere. (**c**) Long-term stability of PSCs with and without CeO_2_@MoS_2_ interlayer under ambient conditions.

To elucidate the charge-carrier dynamics of NiO_x_ NPs incorporating CM NCs, TPV and TPC measurements were conducted to evaluate the carrier recombination and extraction processes. Figure 8a,b present the TPV and TPC of devices containing 0 vol% and 2 vol% CM NCs, while the corresponding carrier lifetimes and extraction times are summarized in Table 2. The recombination lifetime increased from 4.18 ms to 5.06 ms upon introducing 2 vol% CM NCs into the NiO_x_ NPs layer, consistent with the previously observed enhancements in J_SC_ and FF. In addition, the radiative and non-radiative recombination behaviors were further analyzed by examining the dependence of V_OC_ and J_SC_ on light intensity [61,62]. An ideality factor (n) approaching 1 indicates that radiative recombination becomes the dominant recombination pathway, suggesting reduced trap-assisted non-radiative recombination and improved interfacial quality. Similarly, an α value close to 1 suggests minimal non-radiative recombination losses. The extracted ideality factors and α values are listed in Table 2. As shown in Figure 8c,d, the ideality factor decreases from 1.91 to 1.76 upon incorporation of 2 vol% CM NCs, indicating reduced radiative recombination and consequently lower efficiency losses compared with the control device. Moreover, the shelf stability of the device was evaluated by storing it in the dark under an N_2_ atmosphere. After 600 h, the device retained, on average, ~85% of its initial PCE (Figure 9a). The operational stability was further evaluated by maximum power point (MPP) tracking under continuous illumination using an LED solar simulator (LSH-7320, Taichung City, Taiwan) in a nitrogen atmosphere, with the PCE periodically recorded, as shown in Figure 9b. Compared with the control device, the modified device exhibits markedly enhanced operational stability. In addition, the shelf stability of the unencapsulated devices was investigated by storing them in the dark under ambient air conditions (30 °C, RH 40%), as presented in Figure 9c. After 56 h of storage, the devices retained approximately 90% of their initial PCE, indicating good environmental stability. This improved stability is attributed to defect-state reduction and suppression of deep trap densities from CM NCs incorporation within the NiO_x_ nanoparticle layer.

## 4. Conclusions

In summary, we demonstrated an effective interfacial engineering strategy that simultaneously achieved defect passivation and efficient hole extraction in PSCs by incorporating CM NCs at the NiO_x_/MeO-2APCz interface. The initial PCE of 17.01%. was measured for the reference device. The device performance was substantially enhanced upon incorporation of CM NCs interlayer between NiO_x_ and MeO-2PACz. The PCE was strongly dependent on the concentration of CM NCs, and an optimal loading condition was systematically identified. At CM NCs concentrations of 1 vol% and 2 vol%, the PSCs exhibited boosted PCEs of 17.50% and 17.93%, respectively, whereas excessive loading (4 vol%) led to performance degradation (17.07%) due to increased charge-transport resistance and excessive interfacial thickness. The enhanced device performance at the optimal CM NCs concentration is attributed to the synergistic effects of defect-rich MoS_2_ and oxygen vacancies induced by CeO_2_, which suppress the carrier recombination lifetime and reduce hole extraction time. Furthermore, the reduced ideality factor observed for devices incorporating 2 vol% CM NCs confirms the effective suppression of trap-assisted non-radiative recombination and improved interfacial charge transport. These findings show the potential of CM NCs as an effective interfacial modifier for improving PSC performance by simultaneously regulating defect states and charge-carrier dynamics, thereby offering a promising pathway towards low-cost, efficient, and stable perovskite solar cells for future solar energy harvesting applications.

## Figures and Tables

**Table 1:** Photovoltaic parameters of PSCs incorporating different CM NCs concentrations.

CM NCs Amount (vol%)	Type	V_OC_ (V)	J_SC_ (mA/cm^2^)	FF (%)	PCE (%)
0	Champion	1.12	19.14	79.09	17.01
	Average ± SD	1.12 ± 0.01	18.79 ± 0.39	76.59 ± 4.96	16.14 ± 1.02
1	Champion	1.12	19.84	78.63	17.50
	Average ± SD	1.10 ± 0.01	19.38 ± 0.45	76.71 ± 3.01	16.38 ± 0.97
2	Champion	1.15	19.65	79.48	17.93
	Average ± SD	1.14 ± 0.01	19.56 ± 0.14	78.07 ± 1.81	17.40 ± 0.49
4	Champion	1.12	19.55	78.24	17.07
	Average ± SD	1.10 ± 0.01	18.54 ± 0.92	71.80 ± 6.10	14.71 ± 1.55

**Table 2:** Charge-transport and recombination parameters of PSCs incorporating CM NCs.

CM NCs Amount (vol%)	Recombination Time (ms)	Extraction Time (μs)	Ideality Factor n	*α*
0	4.18	0.61	1.91	0.99
2	5.06	0.54	1.76	0.99

## Data Availability

Data are contained within the article.
